# Metastatic Melanoma of Uncertain Primary with 5-Year Durable Response after Conventional Therapy: A Case Report with Literature Review

**DOI:** 10.1155/2018/7289896

**Published:** 2018-05-31

**Authors:** Jomjit Chantharasamee, Jitsupa Treetipsatit

**Affiliations:** ^1^Division of Medical Oncology, Department of Medicine, Faculty of Medicine, Siriraj Hospital, Mahidol University, Bangkok, Thailand; ^2^Department of Pathology, Faculty of Medicine, Siriraj Hospital, Mahidol University, Bangkok, Thailand

## Abstract

A 51-year-old Thai woman presented with bilateral leg edema and painful left inguinal mass for 6 months. Physical examination revealed matted bilateral inguinal lymph nodes up to 9 cm in size. Otherwise, physical examinations including skin were unremarkable. The result of the lymph node incisional biopsy is consistent with that of metastatic melanoma. The extensive investigation demonstrated multiple intra-abdominal and inguinal lymph nodes without detectable primary tumor. Palliative radiation and conventional chemotherapy were prescribed. The CT scan between treatments showed that the response was stable disease, but the following CT scan demonstrated a gradual decrease in size from August 2012 to November 2017 including the lesions outside radiation fields. Moreover, she developed vitiligo during a follow-up visit. The previous data reported the median overall survival among the patients who were treated with conventional chemotherapy ranging from 9.1 to 9.3 months and whose 5-year survival was less than 10%. This case represented a metastatic melanoma of unknown primary who achieved a durable response by conventional treatment. The clinical features including nodal-only disease, vitiligo, and abscopal effect of radiation were considered to be the favorable factors.

## 1. Introduction

Malignant melanoma is an uncommon skin malignancy accounting for about 4% of skin cancer [1, 2]. An incidence rate of melanoma is increasing worldwide but varies between different studies ranging from 0.3 to 3.6% [1] depending on the predominant skin type and geographical location. The *prevalence* for men and women varies with the *highest prevalence* at the *fifth decade* of age [2, 3]. Malignant melanoma of unknown primary (MUP) was reported to be 2–2.4% of melanoma [2, 4]. Compared to the other areas, Asian populations have a significantly lower incidence rate that was estimated about 0.2 to 0.5 per 100,000 patient-years; this incidence rate is mostly of melanoma of known primary (MKP), so that makes MKP in Asian population extremely rare [1, 2]. Most of the literatures in the different geographic regions demonstrated better overall survival of MUP than of MKP [2, 5, 6]. A 5-year overall survival of MUP before the era of the immune checkpoint inhibitor was reported to range from 8 to 18% [3, 4, 7]. The most common metastatic site is the lymph node and GI tract [2, 3]. Many hypotheses were documented in relation to the etiology of MUP including spontaneous regression of primary melanoma, undiagnosed excised melanoma, small primary in the visceral site, and primary melanoma in the lymph node [2, 8, 9]. This study was aimed at reporting a patient with metastatic melanoma of uncertain primary who achieved durable response longer than expected after being treated with conventional treatments.

## 2. Case Report

A 51-year-old Thai woman was hospitalized in July 2012 with edema at the left lower extremities and painful left inguinal mass for 6 months.

Physical examination revealed matted bilateral inguinal lymph nodes up to 9 cm in size with hard consistency, erythema, and tenderness without fluctuation or ulcer. Marked swelling at both lower extremities was observed. There was no other superficial lymphadenopathy. Otherwise, physical examinations were normal.

**Figure 1:**
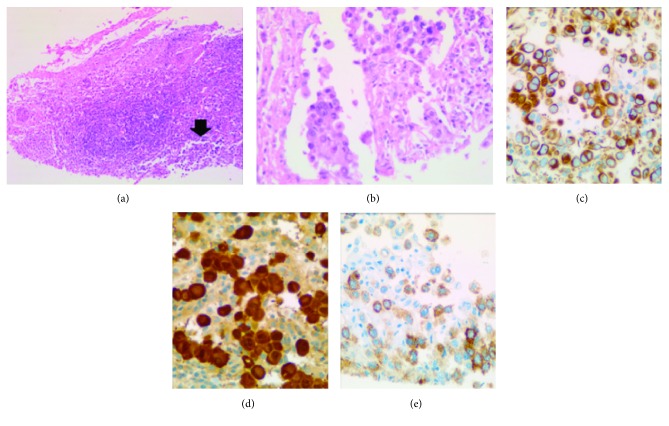
Metastatic malignant melanoma in the left inguinal lymph node: (a) H&E at ×40 and (b) H&E at ×400. The tumor cells are positive for vimentin (c), S100 (d), and HMB-45 (e).

Incisional biopsy of the left inguinal lymph node revealed metastatic round cell tumor which is immunohistochemistry positive for vimentin, S100, and HMB-45. The immunophenotype is consistent with malignant melanoma (Figure 1).

**Figure 2:**
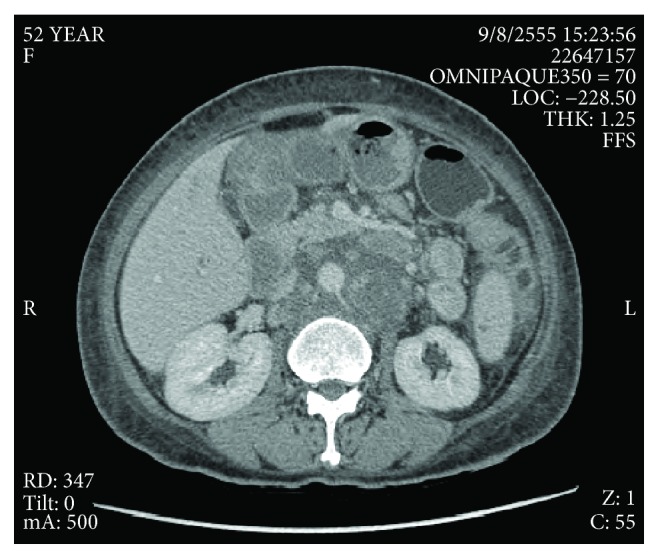
Abdominal CT scan at the time of diagnosis demonstrated matted paraaortic nodes.

**Figure 3:**
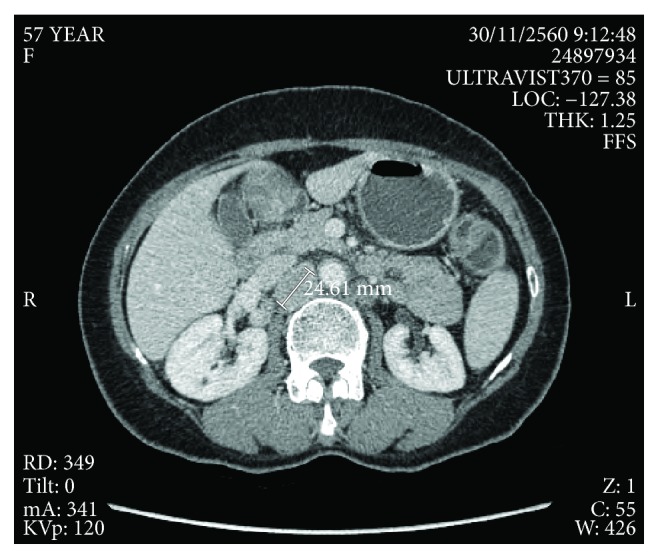
The following CT scan revealed a marked decrease in size of intra-abdominal lymph nodes.

Therefore, primary tumors in the lower extremities, abdominal cavity, and anogenital organ were suspected. By complete skin examination, no cutaneous lesion was identified. Ophthalmoscopy, gastroscopy, colonoscopy, and cystoscopy were completely normal. Genital and pelvic examinations did not show any evidence of lesion. She denied previous abnormal or removal of cutaneous lesion. Computer tomography of the whole abdomen showed multiple enlarged lymph nodes throughout the abdominal and pelvic cavity up to 9.5 cm, along with compression of both iliac veins without an organ-specific lesion (Figure 2). CT chest was unremarkable. The patient was diagnosed with metastatic melanoma of unknown primary. The molecular testing had not been done due to the patient's reimbursement issue, and the specimen was poor in quality for further testing. During the investigation, she developed severe pain requiring high-dose opioid, so she has undergone 20 Gy of palliative radiotherapy for bilateral inguinal lymph nodes. Despite radiotherapy, the remaining tumors were up to 7.4 cm based on the CT scan. For the subsequent systemic therapy, according to a national reimbursement policy, she could not access an immune checkpoint inhibitor or targeted drug. Chemotherapy was prescribed with carboplatin (AUC5) and paclitaxel 175 mg/m^2^ for 6 cycles. After completion of the planned chemotherapy, the symptom was slightly improved. The CT scan at the first 3 months showed that the response was stable disease, but the following CT scan demonstrated a gradual decrease in size over time from August 2012 to November 2017 (Figure 3). During the follow-up period, the patient developed multiple depigmented patches around the lips, trunk, and periorbital and inguinal area, which are typical of vitiligo.

## 3. Discussion

Systemic treatments of metastatic melanoma were developed for many decades since conventional chemotherapy has been considered a standard approach until the emergence of new drugs such as targeted therapy and immunotherapy over the last 10 years. The dramatic and durable response occurred by taking targeted therapy or immunotherapy but not by taking chemotherapy. In the patients who did not have access to those drugs, chemotherapy is a mainstay treatment. Previous data reported the median overall survival among the patients who were treated with conventional chemotherapy ranging from 7.7 to 16 months and whose 5-year survival was 8–18% [4, 6, 7, 10, 11].

This case report represents a patient with metastatic melanoma of unknown primary with durable response by conventional chemotherapy and palliative radiation. When comparing prognosis, MUP tends to have a better prognosis than MKP as reported in previous studies [2, 4–6, 8, 12]. The aforementioned hypotheses of unknown primary including an immunological response that leads to spontaneous regression of primary tumor, unrecognized primary tumor, and the occurrence of malignant ectopic nevus cells in the lymph node itself can be considered the etiology of MUP in this patient, who denied previous removal of cutaneous lesion [2, 8, 9]. The data from Dana-Farber Institute reported that the nodal-only metastasis was an independent favorable prognostic factor of MUP compared to metastasis at other sites [3]. This patient had nodal-only metastasis, which explains that this is a favorable clinical feature. The survival outcome of metastatic melanoma according to the molecular alteration was reported in many literatures. For Asian population, the study reported by Kong et al. [13] showed that KIT mutation was an independent prognostic factor for a shorter survival compared to KIT wild type (30 versus 58 months). Another study by Si et al. [14] reported that BRAF and NRAS mutation was associated with worse overall survival compared to wild-type melanoma (33 versus 53 months). Regarding the effect of palliative chemotherapy, the recent studies showed that the response rate was 10–30% [10, 11, 15] consistent with the response rate of this patient. After completion of chemotherapy, the tumors still had a detectable size as same as previous, but after regular visits, the tumors gradually decreased in size based on the interval CT scan. The possibility of clinical response from the conventional chemotherapy can be explained by the genomic profile. The correlation of somatic mutations with the clinical outcome of melanoma patients treated with carboplatin/paclitaxel either with or without sorafenib was reported by Melissa et al. The patients harboring BRAF mutation and wild type seemed to have longer survival than those harboring NRAS mutation (15.6 versus 5.6 months) in a chemotherapy arm [16]. Another study from Jilaveanu et al. [17] reported the association between marker expression and response to sorafenib plus chemotherapy. This study revealed that the patients with high VEGFR-R2/low ERK1/2 expression correlated with a higher response rate compared to those with low VEGFR-R2/high ERK1/2. However, we could not demonstrate the molecular alteration in our patient due to the unaffordable cost of testing at the time of diagnosis and the poor quality of the 5-year archival specimen. In addition to the chemotherapy treatment, the tumors outside the radiation field also decreased in size which could be either the effect of chemotherapy or the abscopal effect of radiation [18]. This late-response phenomenon could be the effect of the immune response rather than of the chemotherapy itself. According to the effect of radiotherapy, irradiation can induce the host antitumor immune response resulting in the late response after treatment as presented in this case [19]. The hypotheses of presentation of vitiligo concomitant with melanoma by the process of autoimmune-related vitiligo were published in many literatures. The several data-reported specific antigens of melanoma such as TRP1 and TRP2 that were shared by normal melanocytes caused immune response to both melanoma and normal melanocytes [20–22]. In addition, some preclinical evidence supported the role of CD8 T-cell-mediated melanoma in vitiligo. The result from an ex vivo study demonstrated that melanoma cells can be killed by CD8 T-cells taken from a vitiligo lesion and T-cells taken from melanoma that caused apoptosis of melanocytes [21, 23, 24]. Moreover, the report from Becker et al. [25] showed the clonotypically identical T-cell infiltration within the melanoma and vitiligo lesion. All those theories can explain the correlation between melanoma and vitiligo. The evidence supports that the presence of vitiligo may be a favorable prognostic factor for survival theoretically due to the immune mechanism responsible for melanocytic proliferation causing depigmentation of skin and spontaneous regression of primary melanoma which were reported [26–28]. One of the studies reported by Nordlund et al. [28] showed that a 10-year survival rate among patients with nonmetastatic melanoma with vitiligo was 49%. This patient who has vitiligo may benefit from this mechanism in terms of disease control. This patient had a tumor response along with vitiligo later after the complete treatment which was probably from the effect of the immune process. The molecular basis in this patient was not yet known to be a prognostic marker for survival.

## 4. Conclusion

Metastatic melanoma can achieve durable response by conventional chemotherapy and radiotherapy in patients with some clinical characteristics. The presence of the nodal-only disease, vitiligo, and effect of radiation seemed to be the favorable factors for better survival.
